
JOURNAL INFORMATION
==============================
NLM Title Abbreviation: Wirtschaftsdienst
Journal ID: Wirtschaftsdienst
NLM Journal ID: 101086612

Wirtschaftsdienst (Hamburg, Germany : 1949)

ISSN: 0043-6275

ARTICLE INFORMATION
==============================
PMCID: PMC8383020
PMID: 34456386
DOI: 10.1007/s10273-021-2986-2
Article ID: 2986
Article version: 1
Subjects: Zeitgespräch

Versorgungsengpässe während einer Pandemie und was dagegen getan werden kann

Fabra Natalia natalia.fabra@uc3m.es
1
Motta Massimo massimo.motta@upf.edu
2
Peitz Martin martin.peitz@gmail.com
3
1 grid.7840.b 0000 0001 2168 9183 Department of Economics, Universidad Carlos III de Madrid, Calle Madrid, 126, 28903 Getafe-Madrid, Spanien
2 grid.454240.3 Barcelona Graduate School of Economics, Ramon Trias Fargas, 25–27, 08005 Barcelona, Spanien
3 grid.5601.2 0000 0001 0943 599X Department of Economics, Universität Mannheim, Schloss, L7, 68131 Mannheim, Deutschland
Electronic publication date: 2021 Aug 24
Print publication date: 2021
Volume: 101
Issue: 8
First page: 600
Last page: 603
Copyright: © Der/die Autor:in(nen) 2021
License: Open Access: Dieser Artikel wird unter der Creative Commons Namensnennung 4.0 International Lizenz veröffentlicht (https://creativecommons.org/licenses/by/4.0/deed.de).
Open Access wird durch die ZBW — Leibniz-Informationszentrum Wirtschaft gefördert.
License URL: https://creativecommons.org/licenses/by/4.0/

issue-copyright-statement: © The Author(s) 2021

==============================

Die Coronavirus-Krise hat gezeigt, wie wichtig es ist, sich auf Pandemien und andere katastrophale Ereignisse vorzubereiten, die die schnelle Verfügbarkeit einiger lebenswichtiger Güter und Dienstleistungen erfordern. Sich nur auf private Anreize und Marktkräfte zu verlassen, wäre unzureichend. Stattdessen sollten Regierungen und vorzugsweise supranationale Institutionen Maßnahmen zur Vorbeugung, Erkennung und Abschwächung ergreifen. Dies beinhaltet die Einrichtung von Wettbewerbsmechanismen zur Bereitstellung lebenswichtiger Güter, die Festlegung von Rationierungsprotokollen und Unterstützung beim Hochfahren der Produktion nach Eintritt der Krise. Hierbei ist es Aufgabe der öffentlichen Institutionen, die Bereitstellung von lebenswichtigen Gütern in ausreichender Menge und Qualität zu angemessenen Kosten sicherzustellen.

* Dieser Artikel fasst Fabra et al. (2020) zusammen und ist in weiten Teilen eine Übersetzung der VoxEU-Kolumne der Autor:innen mit dem Titel „Building a strategy for resilience“. Martin Peitz dankt der Deutschen Forschungsgemeinschaft (DFG) für finanzielle Unterstützung durch CRC TR 224 (Projekt B05).

Die vielleicht wichtigste Lehre aus der COVID-19-Pandemie ist, dass wir nicht darauf vorbereitet waren. In Teilen Europas und darüber hinaus herrschte während der ersten Welle ein Mangel an Produkten und Dienstleistungen, die für den Umgang mit der Pandemie unerlässlich sind (Gesichtsmasken, Handdesinfektionsmittel, Tests, Schutzkleidung, Beatmungsgeräte, Kapazitäten zur Nachverfolgung usw.).[1] Vermutlich bestand die „Strategie“ (sofern es denn überhaupt eine gab) oder Hoffnung darin, dass – sollte der Bedarf entstehen – eine drastisch gestiegene Nachfrage durch Importe und/oder eine rechtzeitige Versorgung durch Produktion in der Europäischen Union gedeckt werden würde. Die europäische Produktion litt unter fehlender Produktionskapazität und der Unterbrechung von Lieferketten. In einigen Fällen konnten bestimmte Güter nicht produziert werden, weil ein wesentlicher Input aufgrund von Exportbeschränkungen anderer Länder nicht mehr verfügbar war.

Jetzt, da wir die Folgen solcher Engpässe und Unterbrechungen kennen, haben wir gelernt, dass es sich lohnt, auf die nächste Krise vorbereitet zu sein – sei es eine andere Pandemie oder ein Schock ganz anderer Art (wie eine Naturkatastrophe, ein Atomunfall oder ein lähmender Cyberangriff). Die Schlüsselfrage ist, wie man eine Resilienzstrategie entwickelt, die hilft, negative Folgen abzumildern oder gar zu verhindern, auch wenn es keine Uni-

1 Wie die Weltgesundheitsorganisation schreibt, hat „die COVID-19-Pandemie … Defizite in der Vorbereitung auf allen Ebenen aufgezeigt, was den Daten- und Informationsaustausch, die Kommunikation und Nachrichtenübermittlung, die Verteilung von medizinischer Ausrüstung (z.B. persönliche Schutzausrüstung), qualifiziertes Personal, den Umgang mit Reisebeschränkungen und das Testen und Verfolgen von Fällen betrifft.“ (Eigene Übersetzung aus dem Englischen, World Health Organization, 2020, 33).

versallösung gibt. Über die Bereitstellung lebenswichtiger Güter hinaus umfasst eine Resilienzstrategie eine robuste Infrastruktur betreffend Gesundheitssystem, Logistiknetzwerk, Informations- und Kommunikationssysteme sowie Entscheidungsgremien für Notfälle, die Legitimität mit Fachwissen verbinden, und Institutionen, welche die Zusammenarbeit zwischen Ländern oder Regionen fördern.[2]

## Wie zukünftige Krisen entschärft werden können

### Allgemeine Forschungsinfrastruktur und Humankapital

Eine Resilienzstrategie beinhaltet auch den Aufbau und die Pflege einer angemessenen Forschungsinfrastruktur und von Humankapital sowie Investitionen in die Prävention, Früherkennung (zur Abschwächung ihrer Auswirkungen) und den Aufbau der Reaktionsfähigkeit im Fall des Eintretens solcher Ereignisse (National Academy of Medicine, 2016).

Eine geeignete physische Infrastruktur und gut geschulte Arbeitskräfte zeichnen generell ein funktionierendes Gesundheitssystem aus. Während der ersten Welle der COVID-19-Pandemie beruhte der relative Erfolg Deutschlands vermutlich auch auf seinem dichten Netz von Gesundheitseinrichtungen, chemischen und biochemischen Labors sowie auf der großen Zahl von Intensivbetten (ICUs). Die Coronavirus-Krise hat deutlich gemacht, wie wichtig es ist, über genetische Forschungszentren und pharmazeutische Labors zu verfügen, um das Virus zu sequenzieren, seine Entwicklung zu überwachen, einen Impfstoff zu entwickeln und geeignete Medikamente zu finden.[3] Da Forschung und die zugrundeliegende Forschungsinfrastruktur ein öffentliches Gut sind, ist zu erwarten, dass es bei einer privaten Bereitstellung zu einer Unterversorgung kommt, sodass eine öffentliche Unterstützung erforderlich ist.[4]

2 Während wir uns hier auf die direkten (und unmittelbaren) Auswirkungen konzentrieren, bezieht eine auf das Wohl der Gesellschaft ausgerichtete Resilienzstrategie auch die indirekten Auswirkungen mit ein. Dass dies notwendig ist, hat die COVID-19-Pandemie deutlich gezeigt: „COVID-19 betrifft alle Lebensbereiche. Sie erfordert daher eine umfassende Reaktion … In vielen Sektoren fehlten Pläne, um nicht nur die Risiken für die öffentliche Gesundheit, sondern auch die potenziellen sozioökonomischen Auswirkungen der Pandemie abzumildern … Die potenziellen Auswirkungen von Pandemien auf andere Sektoren als das Gesundheitswesen und den Privatsektor sind seit Jahren ein bekanntes Risiko.“ (Eigene Übersetzung aus dem Englischen, World Health Organization, 2020, 33).

3 Aghion et al. (2020) erläutern, wie die US Biomedical Advanced Research and Development Authority (BARDA) die Impfstoffforschung unterstützt. Aghion et al. (2020) und Fabra et al. (2020) schlagen die Schaffung einer europäischen BARDA vor.

4 Eine engere Koordinierung auf EU-Ebene in diesem Bereich wäre ebenfalls wichtig, z.B. könnte das European Research Council oder ein ähnliches Gremium die Bereiche ermitteln, in denen die Forschung zu möglichen Katastrophenereignissen am dringendsten benötigt wird, und Labore abhängig von ihrer Leistung finanzieren.

### Öffentliche Institutionen

Es liegt in der Natur der Sache, dass Krisen externe Effekte auslösen, die einzelne Personen nicht internalisieren und sich, wie im Fall von Pandemien, über Ländergrenzen hinweg ausbreiten. Daher spielen öffentliche Institutionen und die Koordination zwischen ihnen eine Schlüsselrolle bei einer geeigneten Resilienzstrategie.

Staatliche Regulierung hat zum Ziel, risikoreiches Verhalten von Einzelpersonen zu vermindern oder zu verhindern. Öffentliche Institutionen werden auf nationaler und supranationaler Ebene benötigt:

1. unabhängige nationale (oder regionale) Behörden, die mit der Aufgabe betraut sind, Präventivmaßnahmen zu entwickeln und zu implementieren sowie

2. multi- oder internationale Institutionen, die mit der Koordinierung von Maßnahmen betraut sind oder möglicherweise eigene Kompetenzen für das Ergreifen von Präventivmaßnahmen haben.

### Prävention und Krisenmanagement

Früherkennungsinstrumente und Stresstests für kritische Sektoren (z.B. Pharmazeutika, medizinische Versorgung, Versorgungsunternehmen) sollten Teil einer Reihe von Präventionsmaßnahmen sein, die es den Behörden ermöglichen, Ausbrüche zu antizipieren und die Gesellschaft darauf vorzubereiten. Prävention beinhaltet, dass die Bereitstellung lebenswichtiger Güter sichergestellt wird, welche die Menschen während einer Krise benötigen. Zu diesem Zweck sollte eine Resilienzstrategie folgende Komponenten beinhalten:

- Lagerung lebenswichtiger Güter,

- Maßnahmen, die garantieren, dass mit Eintritt einer Krise die Produktion und Versorgung schnell hochgefahren werden und

- klare Rationierungsprotokolle für jene Güter und Zeiträume, in denen die Produktion nicht ausreicht, um die Nachfrage unter Beachtung von Preisobergrenzen zu bedienen.

Bei lagerfähigen Gütern und Vorleistungen stellt sich die Frage, welcher Anteil gelagert werden sollte und welcher Anteil durch hochgefahrene Produktionskapazitäten bereitgestellt werden könnte. Hierbei ist der Zeitfaktor zu berücksichtigen, also insbesondere wie lange es dauert, zusätzliche Produktionskapazitäten zu aktivieren. Bezüglich des Anteils an lebenswichtigen Gütern, der gelagert werden soll, hat der Staat die Möglichkeit, dies selbst zu tun oder diese Dienstleistung von privaten Parteien zu beziehen. Da diese Dienstleistung kontinuierlich erbracht wird, können Standard-Beschaffungspraktiken verwendet werden, begleitet von einer angemessenen Überwachung, die sicherstellt, dass die Vertragspartner die Bestimmungen einhalten. Der Teil der lebenswichtigen Güter und Betriebsmittel, der nicht gelagert wird, sondern bei Ausbruch oder während einer Krise produziert werden muss, erfordert eine gewisse Überkapazität vor dem Schock. Dabei kann es sich um ungenutzte Kapazitäten handeln oder um Kapazitäten, die schnell von ihrer Verwendung in normalen Zeiten auf die Produktion des spezifizierten essenziellen Gutes umgestellt werden können.[5] Da die Bereitstellung von Überkapazitäten auf Strommärkten gang und gäbe ist, untersuchen wir, welche Einsichten, die dabei gewonnen wurden, sich auf unsere Fragestellung übertragen lassen.

## Aus den Erfahrungen des Strommarktdesigns lernen

Strommärkte illustrieren die Notwendigkeit von Überkapazitäten in normalen Zeiten. Die Speicherung von Strom ist aktuell nur beschränkt möglich. Dennoch müssen Stromnachfrage und -angebot jederzeit ausgeglichen werden, auch in Zeiten, in denen keine erneuerbaren Ressourcen zur Verfügung stehen, andere negative Angebotsschocks auftreten oder die Nachfrage aufgrund von extremen Witterungen oder unerwarteten Ereignissen Spitzenwerte erreicht.

Der Preismechanismus kann dies nicht gewährleisten, wenn es Obergrenzen für die Verbraucherpreise gibt, die Verbraucherpreise nicht schnell angepasst werden können oder die Verbraucher:innen nicht schnell auf Preissignale reagieren. Dann muss die Nachfrage rationiert werden. Andernfalls kollabiert das Stromnetz. Die Stromkrise in Texas im Winter 2021 ist ein Beispiel dafür. Ein Wintersturm reduzierte die verfügbare Kapazität, während die Stromnachfrage aufgrund der eisigen Temperaturen in die Höhe schoss, sodass 70 % von Texas ohne Strom blieben.

Angesichts der Schwierigkeiten beim Ausgleich von Angebot und Nachfrage durch aktive Nachfragereduzierung haben Stromregulierungsbehörden Regulierungsmechanismen (die sogenannten Kapazitätsmechanismen) eingeführt, um zu gewährleisten, dass hinreichende Erzeugungskapazitäten geschaffen werden, auch wenn ein Teil davon nur bei selten auftretenden Ereignissen genutzt würde. Die Parallele zur Notwendigkeit, die Versorgung mit bestimmten Gütern in Notsituationen zu gewährleisten, ist offensichtlich, ebenso wie die Tatsache, dass die Nichtbereitstellung solcher Güter hohe gesellschaftliche Kosten verursachen würde.

In der Praxis werden unterschiedliche Kapazitätsmechanismen angewandt, aber sie haben alle ein gemeinsames Merkmal: Die Wahl der Kapazität wird nicht dem Markt überlassen. Vielmehr entscheidet der Regulierer, wie viel Kapazität zur Verfügung gestellt werden muss, und ein Wettbewerbsmechanismus bestimmt, welche Unternehmen diese Kapazität bereitstellen und wie sie dafür entlohnt werden (Fabra, 2018). Die Vorhersage über die notwendige Kapazität ist schwierig. In der Elektrizitätswirtschaft erfordert sie die Vorhersage des zukünftigen Strombedarfs und -angebots (die „bekannte Unbekannte“ sind), aber in unserem Kontext erfordert sie die Einschätzung der Natur möglicher zukünftiger Krisen und eine Entscheidung, für welche dieser Krisen Vorkehrungen getroffen werden sollen (die durchaus „unbekannte Unbekannte“ sein können).

Wir überspringen die Frage, wie genau die Kapazität zu bestimmen ist, und betrachten zwei Aspekte des Marktdesigns. Eine wichtige Gestaltungsdimension ist die Frage, ob Kapazitätsmärkte zentral oder dezentral organisiert sein sollen. Bei zentralen Mechanismen richtet die Regulierungsbehörde eine zentrale Auktion ein, die dazu dient, die Kraftwerke zu bestimmen, die sich verpflichten, die Versorgungssicherheit zu gewährleisten, sowie den Preis, zu dem sie dies tun werden. Bei dezentralen Mechanismen erlegt die Regulierungsbehörde den Stromeinzelhändlern eine Kapazitätsverpflichtung auf, die Kapazitätsgutschriften von Kapazitätsanbietern kaufen müssen, entweder auf bilateraler Ebene oder über Börsen. Die Nichteinhaltung dieser Verpflichtung wird durch Geldstrafen geahndet. Denken Sie an die Parallele im Fall von Grippeimpfstoffen. Bei einem zentralen Mechanismus würde die Regierung alle für die gesamte Bevölkerung benötigten Impfstoffe beschaffen, während bei einem dezentralisierten Mechanismus die Regulierungsbehörde diese Verpflichtung den Krankenkassen auf Basis ihrer Versicherten oder den Krankenhäusern entsprechend dem Bevölkerungsanteil in ihrem Einzugsgebiet auferlegen würde.

Typischerweise führen Überlegungen zur Nachfragemacht und Risikoverteilung dazu, dass zentrale Mechanismen zu empfehlen sind. Im Gegensatz dazu könnten Informationsaspekte den dezentralen Ansatz begünstigen. Ein Nachteil dezentraler Systeme ist die Notwendigkeit, ein Sanktions- und Überwachungssystem einzurichten (in Fällen, in denen Verstöße ex ante aufgedeckt werden können), um Nichteinhaltung zu vermeiden. Wenn Versäumnisse erst bei Eintritt eines Schocks aufgedeckt werden, sind Sanktionen möglicherweise unglaubwürdig

5 Wie die National Academy of Medicine (2016, 77) bemerkt, können im Falle einer Pandemie „freie Produktionskapazitäten benötigt werden, um die Massenproduktion von Produkten sowie das Testen von Prüfpräparaten zu ermöglichen“ (Eigene Übersetzung aus dem Englischen).

angesichts der Höhe der Sanktionen, die in einigen Fällen erforderlich sein könnten, damit das Anreizsystem funktioniert und angesichts der Art der beteiligten Akteure – wäre es beispielsweise glaubwürdig, ein Krankenhaus zu bestrafen, das versäumte, während einer Pandemie lebenswichtige Vorräte bereitzuhalten?

Ein weiterer wichtiger Aspekt der Ausgestaltung ist die Frage, ob Kapazitätsmechanismen marktweit sein sollten (d. h. alle bestehenden Unternehmen sollten Anspruch auf Kapazitätszahlungen haben) oder ob sie auf eine Teilmenge von Unternehmen ausgerichtet sein sollten (z. B. nur auf neue Anlagen, nur auf Anlagen in einer bestimmten Region, nur auf bestimmte Technologien usw.). Der Hauptvorteil von marktweiten Mechanismen besteht darin, dass sie die Unternehmen besser auswählen, die bei der Bereitstellung von Kapazitäten effizienter sind. Allerdings müssen marktweite Kapazitätsmechanismen nicht die aus Sicht der Verbraucher:innen kostengünstigste Art der Kapazitätssicherung darstellen. Bei einer Versorgung in Krisenzeiten kann es von Vorteil sein, nur solche Unternehmen zuzulassen, die der Staat bei Nichterfüllung glaubwürdig sanktionieren kann und deren verwendete Technologie als zuverlässig eingestuft wird.

Basierend auf den Erfahrungen mit Stromkapazitätsmärkten können dafür entwickelte Kapazitätmechanismen Teil einer Resilienzstrategie werden. Hierbei ist zu berücksichtigen, dass das Spektrum möglicher Krisen sehr breit ist. Wir sind der Ansicht, dass als Ausgangspunkt fokussierte, zentrale Mechanismen wie beispielsweise ein System strategischer Reserven, die über Wettbewerbsmechanismen zu beschaffen sind, in Betracht gezogen werden sollten, um die Versorgung mit wesentlichen Gütern und Dienstleistungen zu gewährleisten.

Wenn es sich um „Allzweckprodukte“ handelt, können Kapazitätsmechanismen ex ante – also vor Eintreten des Schocks – eingerichtet und während der Krise angepasst werden, wenn sich die Kapazitäten als unzureichend erweisen. Beispiele für solche Allzweckprodukte sind medizinische Gesichtsmasken, Schutzanzüge, Desinfektionsmittel (antiviral, antibakteriell, antimykotisch), medizinischer Sauerstoff, Infusionslösung, sterile Spritzen, Krankenhausbetten. Andere unentbehrliche Produkte sind spezifisch im Gebrauch und müssen so schnell wie möglich entwickelt werden, wenn der Schock eingetreten ist. Beispiele für diese spezifischen essenziellen Produkte sind Impfstoffe, Medikamente und Testkits. Parallel zur Entwicklung dieser Produkte müssen Produktionskapazitäten aufgebaut werden. Kapazitätsmechanismen, die Anreize für die tatsächliche Produktion hochwertiger Produkte beinhalten, können dabei helfen, Innovationen und Produktionsanreize zu stimulieren (Ockenfels, 2021).Die COVID-19-Pandemie lehrt uns, dass Krisenvorsorge eine wichtige Aufgabe staatlichen Handelns ist (vgl. auch Bénassy-Quéré et al., 2020). Die Wirtschaftswissenschaften können den politischen Entscheidungstragenden dabei helfen, eine geeignete Resilienzstrategie zu entwickeln.

Prof. Dr. Natalia Fabra ist Professorin für Volkswirtschaftslehre an der Universidad Carlos III in Madrid, Spanien.

Prof. Dr. Massimo Motta ist Forschungsprofessor an der ICREA-Universitat Pompeu Fabra und an der Barcelona Graduate School of Economics in Barcelona, Spanien.

Prof. Dr. Martin Peitz ist Professor für Volkswirtschaftslehre an der Universität Mannheim und Direktor des Mannheim Centre for Competition and Innovation (MaCCI) in Mannheim.


Literatur

Aghion, P., S. Amaral-Garcia, M. Dewatripont und M. Goldman (2020), How to strengthen European industries’ leadership in vaccine research and innovation, VoxEU, 1. September.
Bénassy-Quéré, A., R. Marimon, P. Martin, J. Pisani-Ferry, L. Reichlin, D. Schoenmaker und B. Weder di Mauro (2020), Repair and reconstruct: A recovery initiative, VoxEU, 20. April.
Fabra N A primer on capacity mechanisms Energy Economics 2018 75 323 335 10.1016/j.eneco.2018.08.003
Fabra, N., M. Motta und M. Peitz (2020), Preparing for the next crisis: How to secure the supply of essential goods and services, CEPR Policy Insight, 106.
National Academy of Medicine (2016), The Neglected Dimension of Global Security: A Framework to Counter Infectious Disease Crises, The National Academies Press.27336117
Ockenfels, A. (2021), Marktdesign für eine resiliente Impfstoffproduktion, Perspektiven der Wirtschaftspolitik, im Erscheinen.
World Health Organization (2020), World in disorder, Global Preparedness Monitoring Board Annual Report 2020.
